# Utilization of sodium-glucose cotransporter 2 inhibitors on dry eye disease severity in patients with type 2 diabetes mellitus

**DOI:** 10.7150/ijms.88720

**Published:** 2023-10-09

**Authors:** Yen-Po Yao, Po-Jen Yang, Chia-Yi Lee, Jing-Yang Huang, Shun-Fa Yang, Hung-Yu Lin

**Affiliations:** 1 Institute of Medicine, Chung Shan Medical University, Taichung, Taiwan.; 2 Department of Ophthalmology, Show Chwan Memorial Hospital, Changhua, Taiwan.; 3 Department of Family and Community Medicine, Chung Shan Medical University Hospital, Taichung, Taiwan.; 4 School of Medicine, Chung Shan Medical University, Taichung, Taiwan.; 5 Department of Ophthalmology, Nobel Eye Institute, Taipei, Taiwan.; 6 Department of Ophthalmology, Jen-Ai Hospital Dali Branch, Taichung, Taiwan.; 7 Department of Medical Research, Chung Shan Medical University Hospital, Taichung, Taiwan.; 8 Department of Post-Baccalaureate Medicine, College of Medicine, National Chung Hsing University, Taichung, Taiwan.; 9 Department of Optometry, Chung Shan Medical University, Taichung, Taiwan.

**Keywords:** dry eye disease, SGLT2 inhibitors, epidemiology, diabetes mellitus, severity

## Abstract

Sodium-glucose cotransporter 2 (SGLT2) inhibitors have protective effects against various systemic diseases and neoplasms. This retrospective cohort study evaluated the severity of dry eye disease (DED) in patients with type 2 diabetes mellitus (T2DM) who were treated with SGLT2 inhibitors. Data were obtained from the National Health Insurance Research Database of Taiwan. Patients with T2DM who were treated with SGLT2 inhibitors were assigned to the SGLT2 group. Each patient in the SGLT2 group was matched to two individuals with T2DM who had not used SGLT2 inhibitors, constituting the control group. The primary outcomes were the development of DED and severe DED. A diagnosis of severe DED was indicated by the usage of cyclosporine. Cox proportional hazard regression was applied to yield adjusted hazard ratios (aHR) and 95% confidence intervals (CIs). In the SGLT2 group, 1864 new DED events and 147 severe DED events were recorded. Conversely, 4367 new DED events and 392 severe DED events were recorded in the control group. The incidence (aHR: 0.858, 95% CI: 0.811-0.908, *p* = 0.0010) and severity (aHR: 0.652, 95% CI: 0.481-0.777, *p* = 0.0006) of DED were significantly lower in the SGLT2 group than the control group after adjusting for multiple covariates. In subgroup analyses, the incidence and severity of DED were significantly lower in patients younger than 60 years old who were treated with SGLT2 inhibitors than in their older counterparts (*p* = 0.0008 and 0.0011, respectively). In conclusion, utilization of SGLT2 inhibitors in the T2DM population could reduce both the incidence and severity of DED.

## Introduction

Type 2 diabetes mellitus (T2DM) is a widespread systemic disease, affecting approximately 10% of the global population 1, 2. The primary treatments for T2DM are insulin injection and oral medication administration 3. Of the available antidiabetic medications, sodium-glucose cotransporter 2 (SGLT2) inhibitors have gained attention for their effectiveness in controlling hyperglycemia 4, 5. A relevant study revealed a 0.71% reduction in glycated hemoglobin levels when SGLT2 inhibitors were added to dipeptidyl peptidase-4 inhibitor monotherapy 6.

SGLT2 inhibitors offer therapeutic benefits in addition to hyperglycemia reduction for patients with T2DM 4. The usage of SGLT2 inhibitors on mice with diabetes decreased the risk of myocardial infarction by suppressing cardiomyocyte autophagy 7. These inhibitors were also associated with a reduced rate of chronic heart failure progression 8. Moreover, for patients with T2DM who have chronic kidney disease, SGLT2 inhibitors were shown to lower glomerular capillary hyperfiltration and thereby reduce physical stress on the filtration barrier 9. In addition to their role in managing hyperglycemia and heart-related problems, SGLT2 inhibitors may offer protective effects against other diseases.

Studies have also reported the influence of SGLT2 inhibitors on ocular diseases 10, 11. Although these inhibitors have been suggested to lower the risk of diabetic retinopathy, some evidence has been collected from animal experiments, and a firm consensus has yet to be reached 12-15. Other studies have demonstrated a decrease in the incidence of dry eye disease (DED), an inflammatory ocular disorder characterized by dryness and irritation, under the use of SGLT2 inhibitors 16, 17. However, no study has yet evaluated the correlation between SGLT2 inhibitors and DED severity.

Consequently, our study aimed to evaluate the possible correlation between the use of SGLT2 inhibitors and DED severity. This investigation relied on data from the National Health Insurance Research Database (NHIRD) of Taiwan. Risk factors potentially related to DED development and severity was accounted for in the statistical analyses.

## Materials and Methods

### Data Source

This study adhered to principles outlined in the Declaration of Helsinki and its subsequent amendments. It received approval from both the National Health Insurance Administration of Taiwan and the Institutional Review Board of Chung Shan Medical University Hospital (Project code: CS1-20108). The requirement for written informed consent was waived by these institutions. The NHIRD of Taiwan served as the data source for this study. The database includes insurance claims data from Taiwan's health insurance system and encompasses medical records for approximately 23 million Taiwanese residents for the period January 1, 2000, to December 31, 2020. The NHIRD of Taiwan contains diagnostic codes from both the *International Classification of Diseases Ninth Revision* (*ICD-9*) and the *International Classification of Diseases Tenth Revision* (*ICD-10*). Additionally, it contains variables such as age, sex, education level, place of residence, imaging examination codes, laboratory examination codes, types of medical departments, procedure codes, surgical codes, and international anatomical therapeutic chemical (ATC) codes for medications.

### Patient Selection

**Figure 1:**
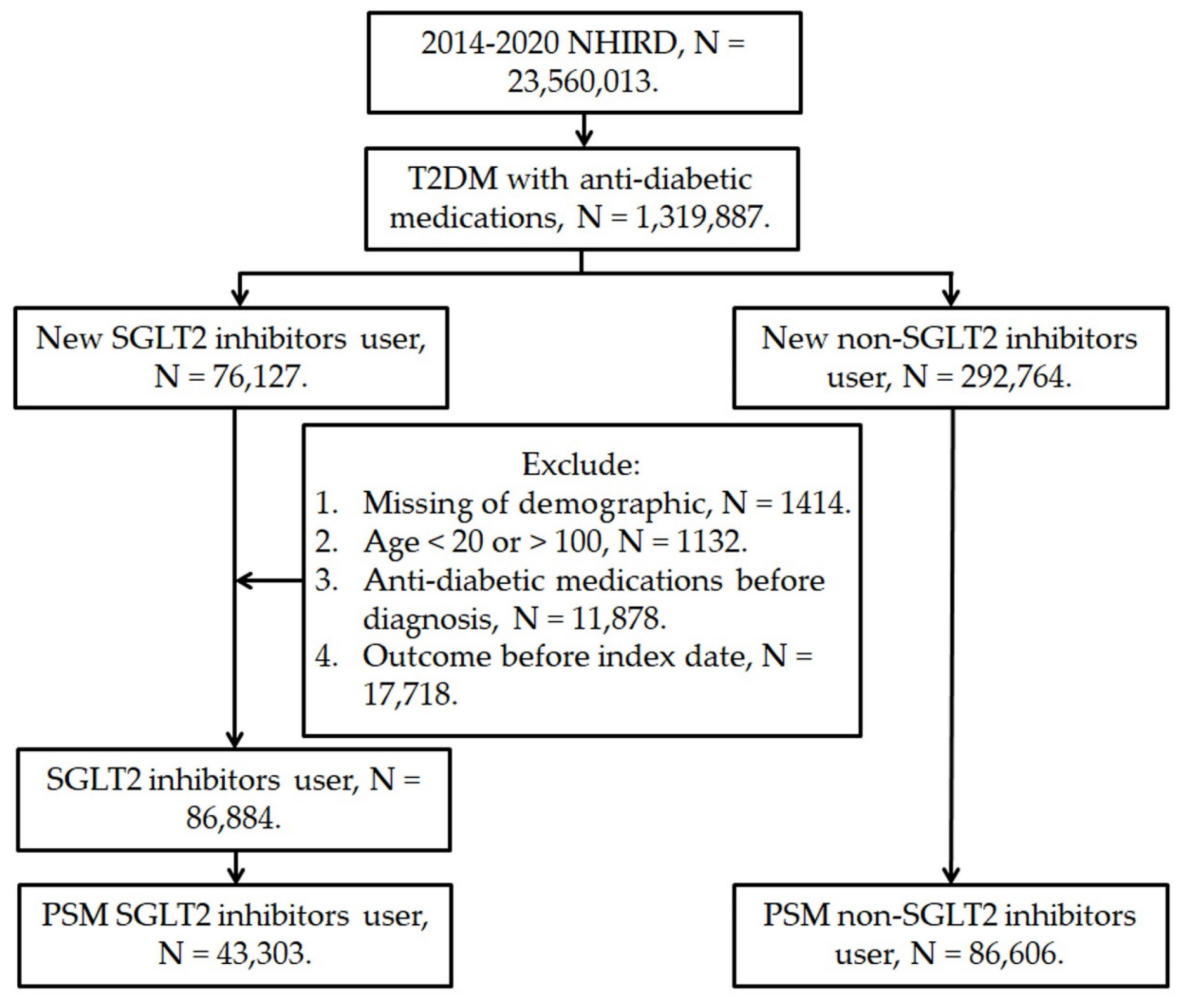
The flowchart of participant selection. NHIRD: National Health Insurance Research Database, N: number, T2DM: type 2 diabetes mellitus, SGLT2: sodium-glucose cotransporter 2, PSM: propensity score-matching.

This retrospective cohort study enrolled patients with T2DM receiving SGLT2 inhibitor treatment if they fulfilled these conditions: (1) they had received a diagnosis of T2DM based on *ICD-9* or *ICD-10* codes between 2014 and 2019; (2) they underwent a minimum of 3 months of follow-up care in either an internal medicine or family medicine department; and (3) they were treated with an SGLT2 inhibitor—including dapagliflozin, canagliflozin, empagliflozin, and ertugliflozin—as identified by ATC codes. The index date for this study was set as the date 6 months after the initiation of SGLT2 inhibitor treatment. In addition, these exclusion criteria were applied: (1) the database contained incomplete demographic data; (2) the patient was prescribed an antidiabetic medication before they received their T2DM diagnosis; (3) the patient was younger than 20 years or older than 100 years; and (4) DED developed before the index date. For comparative purposes, this study also collected a control group of patients with T2DM who had not received an SGLT2 inhibitor. We matched the SGLT2 inhibitor group to the control group by using a PSM procedure that adjusted for demographic, systemic, and medication-related covariates in a 1:2 ratio. A total of 43,303 individuals in the SGLT2 inhibitor group and 86,606 individuals in the control group were selected (Figure 1).

### Primary Outcome Measurement

The primary outcome in this study was the development of DED, which was indicated by the following criteria: (1) assignment of DED-related *ICD-9* or *ICD-10* diagnostic codes; (2) performance of either a tear break-up time examination or a Schirmer test on or before the day of DED diagnosis, as revealed by procedure codes; (3) prescription of artificial tears following DED diagnosis, as indicated by ATC codes; and (4) diagnosis of DED by an ophthalmologist. The severe DED in this study was defined as the complement of above DED criteria plus the application of cyclosporine according to the ATC codes. If a patient did not receive cyclosporine therapy, the patient would be regarded as DED but not severe DED. Patients in the study were followed until either a DED diagnosis, withdrawal from the National Health Insurance program, or December 31, 2020.

### Demographic and Systemic Covariate Enrollment

Multiple demographic data and systemic diseases were adjusted for in our multivariable model to reduce their influence on the outcome of DED development: age, sex, level of urbanization, hypertension, coronary heart disease, hyperlipidemia, cerebrovascular accidents, kidney disease, rheumatoid arthritis, systemic lupus erythematosus, Sjogren syndrome, and ankylosing spondylitis. The existence of these covariates was determined from the related codes in the NHIRD. To ensure that the systemic morbidities had persisted long enough to influence DED development, only those that been recorded for more than 2 years before index date were accounted for.

### Statistical Analysis

SAS version 9.4 (SAS Institute, Cary, NC, USA) was used for statistical analyses. The statistical significance threshold was set at *p* < 0.05. Any *p*-value less than 0.0001 is presented as *p* < 0.0001.

## Results

The baseline characteristics of the following two study groups are presented in Table 1: those treated with SGLT2 inhibitors and a control group. The groups had similar age distributions, with no significant differences discovered. The number of male participants was 28,410 in the SGLT2 group and 56,820 in the control group, the percentage of men was nonsignificantly different between the two groups. The distributions of covariates—including urbanization level, systemic factors, and types of antidiabetic medications used—were also similar in the two groups, as ensured by the propensity score matching (PSM) procedure (all absolute standardized differences < 0.1; Table 1).

Over the course of a follow-up interval of up to 5 years, 1864 and 4367 new DED events were recorded in the SGLT2 and control groups, respectively, whereas 147 and 392 severe DED events were recorded (Table 2). The incidence (adjusted hazard ratio [aHR]: 0.858, 95% CI: 0.811-0.908, *p* = 0.0010) and severity (aHR: 0.652, 95% CI: 0.481-0.777, *p* = 0.0006) of DED were significantly lower in the SGLT2 group than in the control group after adjusting multiple demographic and systemic covariates (Table 2). Other factors that correlated with the development of DED included advanced age, female sex, Sjogren's syndrome, and ankylosing spondylitis (all *p* < 0.05; Table 3).

Subgroup analyses comparing patients with T2DM under 60 years of age who received SGLT2 inhibitors with their counterparts aged 60 years and older demonstrated a significantly lower incidence (*p* = 0.0008) and severity (*p* = 0.0011) of DED in the younger patients (Table 4). However, no significant sex-based differences in DED incidence or severity were discovered (both *p* > 0.05; Table 4).

## Discussion

In this study, the incidence of DED development was significantly lower in patients with T2DM who received SGLT2 therapy than in the control group, according to the multivariable analysis. The incidence of severe DED was lower in the SGLT2 group than in the control group. Patients with T2DM under the age of 60 years who received SGLT2 inhibitors were less likely to develop DED and have severe DED than were those 60 years and older.

Beyond blood sugar control, SGLT2 inhibitors have protective effects on various systemic diseases and neoplasms 18-20. Specifically, the occurrence and progression of coronary heart disease can be retarded by the use of SGLT2 inhibitors 4. A relevant study indicated that these inhibitors can mitigate microvascular damage and endothelial dysfunction in cardiac ischemic injury 21. In addition, major CHD events and related mortality were found to be less likely in patients with T2DM who received SGLT2 inhibitors 22. Patients with congestive heart failure, whether with preserved or reduced ejection fraction, were found to exhibit slower deterioration when they did rather than did not receive SGLT2 inhibitors 8. In addition to cardiovascular benefits, SGLT2 inhibitors have protective effects on kidney function. Specifically, in patients with T2DM, SGLT2 inhibitors were discovered to reduce elevated serum creatinine levels, lower the rate of macroalbuminuria development, and reduce the likelihood of a need for hemodialysis 23. Regarding its molecule pathways, SGLT2 inhibitors have been associated with both anti-inflammatory and antioxidant effects 24-26. They can suppress the inflammatory process in certain types of cardiomyopathy, potentially through modulation of the HIF-2α signaling pathway 27, 28. Moreover, oxidative stress levels were significantly lower in both in vitro and in vivo models of diabetic kidney disease when SGLT2 inhibitors were applied 29. Concerning DED, this inflammatory disease is associated with increased levels of interleukins and tumor necrosis factor-alpha 30. Elevated levels of reactive oxygen species were observed in the conjunctiva of patients with DED 30. Various anti-inflammatory medications, such as cyclosporine and lifitegrast, are used to treat DED 31. Given the broad protective effects of SGLT2 inhibitors and their specific role in inhibiting the molecule pathways in DED 29, 30, we proposed that the application of SGLT2 inhibitors may decrease both the incidence and severity of DED—a hypothesis supported by the results of this study.

In this study, the use of SGLT2 inhibitors was found to lead to both lower incidence and severity of DED in patients with T2DM. These results align with relevant studies that have examined similar populations treated with SGLT2 inhibitors 16, 17. Additionally, our data suggest that the utilization of SGLT2 inhibitors correlates with lower DED severity, as evidenced by lower usage of cyclosporine in this study's participants. To our knowledge, this is a relatively preliminary finding indicating the protective effects of SGLT2 inhibitors regarding DED severity. Moreover, we excluded patients who had received a diagnosis of DED before the index data to eliminate the influence of pre-existing DED. The Cox proportional hazard regression models used in this study were adjusted for multiple known risk factors for DED development and progression. Consequently, the use of SGLT2 inhibitors may serve as an independent protective factor against high-severity DED. One study indicated that elevated tear film osmolarity and inflammatory responses contribute to high-severity DED 30. Selenoprotein P mitigates DED by lowering oxidative stress 32. Given the relevant anti-inflammatory and antioxidant mechanisms, SGLT2 inhibitors should reduce the likelihood of DED progression and high DED severity in patients with T2DM 23, 29. Our findings highlight this possibility.

Concerning the subgroup analyses, the incidence and severity of DED were significantly lower in the patients with T2DM who were under the age of 60 years and treated with SGLT2 inhibitors than in those who were aged 60 years and older. Limited research has demonstrated this phenomenon. However, SGLT2 inhibitors were reported to reduce blood glucose levels across all ages, with significantly lower levels observed in patients less than 40 years old 33. In one study, researchers attributed the enhanced glycemic control achieved under SGLT2 inhibitors to larger urinary glucose excretion 33. Thus, in younger populations, the use of SGLT2 inhibitors may more effectively suppress hyperglycemia-related inflammation, leading to lower DED incidence and severity. Regarding the effect of SGLT2 inhibitors on different sexes, no conclusive evidence was obtained. SGLT2 inhibitors were found to produce similar heart failure control outcomes in men and women 34. Likewise, the reduction in atrial fibrillation following SGLT2 inhibitor usage was nonsignificantly different between the sexes 35. In this study, the aHRs for DED incidence and severity were not significantly different between the sexes, suggesting that sex may not affect the efficiency of SGLT2 inhibitors.

The global prevalence of T2DM and DED is substantial. In China, T2DM affects 8% of the population, whereas in Europe, its prevalence is 8.8% 2. The highest prevalence of T2DM was found to be that in North America (13%) 2. DED is also pervasive, diagnosed in 5%-50% of individuals aged 40 years or older 36. Among visual display terminal users of all ages, the prevalence of DED can exceed 60% 37. Moreover, T2DM can damage the corneal epithelium, leading to corneal erosion and DED development 38. Because T2DM can lead to DED and both diseases affect a large population, whether SGLT2 inhibitors can be employed as a straightforward strategy to reduce the incidence and severity of DED in patients with T2DM warrants exploration.

This study had several limitations. First, the claimed-database design of our study let the blood sugar level, the duration of both DM and DED, the receipt of refractive surgery, the value of body mass index, the patient-reported symptoms and related questionnaires, the values of DED-related exam including the tear-break up time and Schirmer test, the types of DED, the external eye photography of DED patients, and the treatment outcome of DED become unavailable. Also, since the matching of too many covariates will cause over-fitting of the statistical analysis, we did not match all the immune disorder including thyroid disease in this study. Additionally, the severity of DED was assessed on the basis of the use of cyclosporine. However, some medications for advanced DED, including diquafosol and autologous serum, are self-funded and they are not recorded in the NHIRD. Consequently, some cases of severe DED may have been regarded as mild DED. In addition, although the duration of comorbidities was available for analysis, the severity of these co-morbidities, which could influence DED development and severity, was not. Finally, as nearly all the participants in this study were Asian, the external validity of our study could be limited.

In conclusion, the use of SGLT2 inhibitors in patients with T2DM correlates with significantly lower DED incidence and severity after adjusting for several DED risk factors. Furthermore, this protective effect appears to be stronger in patients under 60 years of age. Consequently, SGLT2 inhibitors might be recommended for patients with T2DM at high risk of DED. Further prospective, large-scale studies are required to evaluate the potential for SGLT2 inhibitors to reduce the numbers of treatments in patients with T2DM with DED.

## Figures and Tables

**Table 1:** The characteristic in SGLT2 group and matched diabetes population

Characters	Control group (N = 86,606)	SGLT2 group (N = 43,303)	ASD
Age			
20-39	10746 (12.41%)	6515 (15.05%)	0.0012
40-49	23590 (27.24%)	12066 (27.86%)	0.0008
50-59	29582 (34.16%)	13864 (32.02%)	0.0017
60-69	18219 (21.04%)	8598 (19.86%)	0.0016
70-79	3763 (4.34%)	1891 (4.37%)	0.0004
≥ 80	706 (0.82%)	369 (0.85%)	0.0005
Sex			
Male	56820 (65.61%)	28410 (65.61%)	0.0000
Female	29786 (34.39%)	14893 (34.39%)	0.0000
Urbanization			
Level 1	21506 (24.83%)	11589 (26.76%)	0.0056
Level 2	28368 (32.76%)	14387 (33.22%)	0.0010
Level 3	16882 (19.49%)	8073 (18.64%)	0.0015
≥ Level 4	19850 (22.92%)	9254 (21.38%)	0.0009
Co-morbidity			
Hypertension	41859 (48.33%)	22744 (52.52%)	0.0245
CHD	5875 (6.78%)	5506 (12.72%)	0.0874
Hyperlipidemia	53466 (61.73%)	29962 (69.19%)	0.0659
Cerebrovascular accident	3347 (3.86%)	1590 (3.67%)	0.0008
Kidney disease	4238 (4.89%)	2960 (6.84%)	0.0437
Rheumatoid arthritis	421 (0.49%)	160 (0.37%)	0.0153
Systemic lupus erythematosus	56 (0.06%)	35 (0.08%)	0.0006
Sjogren syndrome	250 (0.29%)	125 (0.29%)	0.0000
Ankylosing spondylitis	681 (0.79%)	368 (0.85%)	0.0008
Co-medication			
Biguanides	73225 (84.55%)	38640 (89.23%)	0.0077
Sulfonylureas	26345 (30.42%)	19052 (44.00%)	0.0561
Alpha glucosidase inhibitors	3133 (3.62%)	2903 (6.70%)	0.0704
Thiazolidinediones	4362 (5.04%)	4036 (9.32%)	0.0664
dipeptidyl peptidase-4 inhibitor	21935 (25.33%)	18130 (41.87%)	0.0833
Insulin	3969 (4.58%)	3874 (8.95%)	0.0992

N: number, SGLT2: sodium-glucose cotransporter 2, ASD: absolute standard difference, CHD: coronary heart disease

**Table 2:** The risk of dry eye disease between SGLT2 and control groups

Event	Control group	SGLT2 group	P
DED			
Person-months	1715969	877022	
Event	4367	1864	
Crude HR (95% CI)	Reference	0.837 (0.793-0.883)*	
aHR (95% CI)	Reference	0.858 (0.811-0.908)*	0.0010
Severe DED			
Person-months	1715969	877022	
Event	392	147	
Crude HR (95% CI)	Reference	0.645 (0.463-0.759)*	
aHR (95% CI)	Reference	0.652 (0.481-0.777)*	0.0006

DED: dry eye disease, aHR: adjusted hazard ratio, CI: confidence interval * Denotes significant difference between the two groups

**Table 3:** The effect of each parameter on the development of dry eye disease

Parameters	aHR	95% CI	P
SGLT2 inhibitors	0.652	0.481-0.777	0.0010*
Age	1.875	1.013-2.651	0.0007*
Male sex	2.007	1.285-3.428	0.0003*
Urbanization	1.045	0.468-2.364	0.8769
Co-morbidity			
Hypertension	1.232	0.782-1.964	0.6213
CHD	0.942	0.559-1.806	0.6652
Hyperlipidemia	1.367	0.656-2.228	0.5726
Cerebrovascular accident	1.003	0.877-1.380	0.7893
Kidney disease	0.737	0.661-1.482	0.0958
Rheumatoid arthritis	1.357	0.918-2.408	0.0738
Systemic lupus erythematosus	0.998	0.790-1.102	0.3542
Sjogren syndrome	1.495	1.113-2.091	0.0016*
Ankylosing spondylitis	1.534	1.008-2.235	0.0334*

aHR: adjusted hazard ratio, CI: confidence interval, SGLT2: sodium-glucose cotransporter 2, CHD: coronary heart disease * Denotes significant difference between the two groups

**Table 4:** The subgroup analyses for dry eye disease development stratified by age and sex

Parameters	aHR	95% CI	P for interaction
DED			
Age			0.0008*
< 60	0.723	0.576-0.834	
≥ 60	0.920	0.878-0.993	
Sex			0.5381
Male	0.827	0.772-0.874	
Female	0.856	0.825-0.936	
Severe DED			
Age			0.0011*
< 60	0.567	0.404-0.742	
≥ 60	0.715	0.555-0.798	
Sex			0.4076
Male	0.618	0.469-0.753	
Female	0.680	0.507-0.821	

DED: dry eye disease, aHR: adjusted hazard ratio, CI: confidence interval, SGLT2: sodium-glucose cotransporter 2 * Denotes significant difference between the two groups
